# Ceramic Scaffolds for Bone Augmentation: Design and Characterization with SEM and Confocal Microscopy

**DOI:** 10.3390/ma15144899

**Published:** 2022-07-14

**Authors:** Alin Gabriel Gabor, Virgil-Florin Duma, Mihai M. C. Fabricky, Liviu Marsavina, Anca Tudor, Cosmin Vancea, Petru Negrea, Cosmin Sinescu

**Affiliations:** 1 Research Center in Dental Medicine Using Conventional and Alternative Technologies, School of Dental Medicine, “Victor Babes” University of Medicine and Pharmacy of Timisoara, 9 Revolutiei 1989 Ave., 300070 Timisoara, Romania; gabor.alin30@gmail.com (A.G.G.); fabricky@me.com (M.M.C.F.); atudor@umft.ro (A.T.); 2 3OM Optomechatronics Group, Faculty of Engineering, Aurel Vlaicu University of Arad, Str. Elena Dragoi No. 2, 310177 Arad, Romania; 3 Faculty of Mechanics, Polytechnic University of Timisoara, 1 Mihai Viteazu Ave., 300222 Timisoara, Romania; liviu.marsavina@upt.ro; 4 Faculty of Chemistry and Environmental Engineering, Polytechnic University of Timisoara, 6 Vasile Parvan Ave., 300223 Timisoara, Romania; cosmin.vancea@upt.ro (C.V.); petru.negrea@upt.ro (P.N.)

**Keywords:** ceramic biomaterials, bone scaffolds, tissue engineering, apparent porosity, scanning electron microscopy (SEM), confocal microscopy (CM), compressive strength, biocompatibility

## Abstract

Bone scaffolds must fulfil numerous and sometimes contradictory characteristics: biocompatibility, bioactivity, high porosity, and appropriate mechanical strength. To tackle some of these issues, this study has several aims for the development of such scaffolds for dentistry applications: (i) to utilize appropriate materials (ceramics and sponges) and to introduce a novel, potentially performant ceramic material; (ii) to characterize the obtained scaffolds by using a range of methods; (iii) to compare and to correlate the assessment results with the scope to validate them reciprocally. There are two commercially available dental ceramics (i.e., Ceramco iC Natural Enamel (E) and Ceramco iC Natural Dentine (D), (DeguDent GmbH, Hanau-Wolfgang, Deutschland)) that are considered, as well as a new-developed porcelain (ceramic C). To obtain porous structures of scaffolds, each ceramic is introduced in two different sponges: a denser one, green (G) and a less dense one, blue (B). A total of 60 samples are manufactured and divided in six study groups, obtained by combining the above materials: GE, BE, GD, BD, GC, and BC (where the first letter represents the sponge type and the second one the utilized ceramic). Several methods are applied to characterize their chemical composition, as well as their macro- and micro-porosity: X-ray Diffraction (XRD), apparent porosity measurements, scanning electronic microscopy (SEM), and confocal microscopy (CM). The latter two methods image the inner (porous) and the outer/cortical (denser) areas of the samples. The results show a good porosity (i.e., dimensions and uniformity of pores) of around 65% for the final group BC, with satisfactory values of around 51% for BD and GC. A certain correlation is made between SEM, CM, and the apparent porosity results. The biocompatibility of the new ceramic C is demonstrated. Finally, a necessary trade-off is made with the mechanical strength of the obtained scaffolds, which was also evaluated. From this point of view, Group BD has the highest compressive strength of around 4 MPa, while Group BC comes second, with around 2 MPa. This trade-off between porosity and mechanical strength suggests a choice between Groups BC and BD, which are the best with regard to the porosity and mechanical strength criterium, respectively.

## 1. Introduction

The main objective of tissue engineering (TE) is to develop biological substitutes that maintain, improve, or restore tissue functions [1,2,3,4]. Scaffolds are important for TE because they are capable of providing a healthy environment for the attachment, differentiation, proliferation, and migration of cells both during in vitro culture and in vivo implantation [5,6]. At the beginnings of TE, permanent implants and scaffolds have been made of biocompatible, bioactive, or bioinert (at that moment) materials, with the purpose of replacing tissues and to provide their functions as well. Currently, scaffolds are used to serve as an extracellular matrix, to emulate the morphological and functional characteristics of the tissues that they replace, or to provide an extracellular matrix to replace them [7]. Numerous types of tissues are targeted, including soft ones such as skin, ligament, cartilage, skeletal muscles, as well as hard tissues such as bone or teeth [8,9]. Research that was focused on the biological integration of implant materials within the host tissue has led to developing mechanical and physiological characteristics of biomaterials that are used for scaffolds that are similar to those of native tissues [1].

Scaffolds are porous structures with three-dimensional (3D) configurations that are made of biocompatible materials that can be doped with various cells or materials to stimulate tissue regeneration. Also, supplemental procedures such as laser bio-stimulation can be applied to achieve this goal [10,11]. Studies in TE have been focused on developing new materials, on modifying the composition and microstructure of existing ones, or on studying procedures to enhance tissue formation [12]. An ideal scaffold has properties that allow for the propagation of new blood vessels and cells, as well as for the differentiation of cells. Also, it must have mechanical properties that correspond to the replaced tissue [13], except for biodegradable bone scaffolds. Therefore, the materials that are proposed for scaffolding are selective and are specific for each type of tissue. This is a major challenge that must consider their various limitations in terms of realization, biocompatibility, and optimal integration [14].

The optimal design of biocompatible materials, specifically ceramics for regenerating bone tissue has been a major goal in recent years. Biocompatible scaffolds, acting as temporary guides for cell adhesion and proliferation in a 3D architecture are key components of tissue regeneration along with cells, enzymes, and growth factors. In TE they can be used when a bone defect arises from causes such as trauma, cancers, fractures, or tooth loss, as well as in degenerative pathologies such as osteoporosis [15,16].

Materials that were investigated for healing and bone regeneration include natural or synthetic polymers, ceramics, metals, and composites [17]. The scaffolds design is complex and intriguing at the same time because certain properties may be reproduced for the specific tissue to be regenerated, while all scaffolds must meet biological and structural requirements that are interconnected and often contradictory: (i) they must be made with non-toxic, non-allergenic, and non-inflammatory materials (*biocompatibility*); (ii) they should be able to induce a biological response to the surface when they are implanted, resulting in a physical link between tissue and material (*bioactivity*); (iii) scaffolds must have a *porous structure* (to mimic the extracellular matrix of the tissue they would restore inside the body), able to allow cell penetration, vascularization, and fluid diffusion; and (iv) they should have *appropriate mechanical properties* (i.e., a sufficient mechanical strength) to allow for handling [18]. To obtain a new bone augmentation material of the scaffold type, the literature provides several methods, including CAD/CAM, 3D printing, and foam replication [16]. All of them aim to obtain a structure with a porosity that is close to natural bone. Most scaffolds that are based on bioactive glass ceramic are made using *the foam replication method*, which involves the production of ceramic foams by coating a polymer sponge with a bio-ceramic slurry. In the following phase, the sponge is burnt during a suitable heat treatment, which at the same time sinters the ceramic powders. This type of scaffold has a high porosity, which is unfortunately associated with poor mechanical properties. An alternative method is *the polymer burning method*, where organic fillers, which act as pore formers, are added to the ceramic powders. The organic phase is then removed during sintering [18].

For bone TE, which is the topic of this study, macro porous ceramic glass scaffolds are essential [1,2,3,4], as they act as a temporary guide for cell proliferation. From the point of view of the aspects above (bioactivity, high porosity, appropriate mechanical properties, as well as a resistant and permeable surface, the latter for controllable samples for both in vitro and in vivo applications), the standard construction technique usually has relatively poor results because of the difficulty in handling fragile surfaces. This imposes dedicated training procedures [19].

In dentistry applications, which are the target of this work, there are various traditional methods for bone augmentation: (i) bone addition by autologous bone that is harvested from the patient in a donor area such as the iliac crest, the ribs, or the mandibular branch (which is a traumatic procedure for the patient and requires repeated surgeries); (ii) with a smack that is made up of various animal bone in combination with various ceramic additions, hydroxyapatite, which are introduced into the area where bone addition is required and coated with a collagen or titanium membrane which is fixed through small screws. This latter method is less traumatic but it has several drawbacks: an additional surgery to procure the tissue; a long time to ossify the smack and to have a primary stability, as bone grafts take a long time to heal; increased operative time and cost; donor site morbidity and postoperative pain; increased risk of fracture to the donor site; limited amount of tissue which can be procured; and a high variability in the quality of the harvested bone tissue.

In contrast to the above, the aims of this study are to propose and develop a new, purely ceramic scaffold material for bone TE for dentistry, and to compare it to two other possible materials that have not been used so far for TE (i.e., commercially available biocompatible ceramics). To introduce new materials, it is necessary to characterize them as accurately as possible from a micro- and macro-structural point of view. Therefore, several methods of characterization (commonly used in literature for a new type of material) are utilized to assess the results and thus, to complete this comparison. Their results are correlated in the present study: *X-ray Diffraction (XRD)*, *apparent porosity measurements, SEM, CM*, and *mechanical testing* [20,21,22,23]. There were two preliminary studies that were carried out, the first one on the mechanical aspects of a possible application of such scaffolds [24], and the second one on the physicochemical characteristics and biosecurity profiles that include the two commercially available ceramics that were considered in the present study [25]. The *biocompatibility* of the new proposed ceramic is tested in the present study, and this is another aim.

## 2. Materials and Methods

### 2.1. Samples Preparation

A total of three types of ceramics were used in this study to manufacture scaffold samples. Of these, two of them are commercially available dental ceramics: Ceramco iC Natural Enamel (E) and Ceramco iC Natural Dentine (D), (DeguDent GmbH (a Dentsply Sirona Company), Hanau-Wolfgang, Deutschland). Their compositions include 80–100% Sodium Potassium Aluminosilicate and up to 5% tin oxide [26]. The third ceramic (C) was designed for the purpose, with the chemical composition including kaolin 52%, feldspar 23.5%, and sand 24.5%. The raw materials oxidic composition (weight%), as provided by the suppliers are: quartz sand—97.3% SiO_2_, 0.35% Fe_2_O_3_, 1.0% Al_2_O_3_, 0.3% (Na_2_O + K_2_O), 0.2% (CaO + MgO), 0.07% TiO_2_; feldspar—78.1% SiO_2_, 12.23% Al_2_O_3_, 0.26% Fe_2_O_3_, 3.8% Na_2_O, 3.61% K_2_O, 0.5% MgO, 1.5% CaO; kaoline—49.29% SiO_2_, 35.18% Al_2_O_3_, 0.78% Fe_2_O_3_, 0.14% Na_2_O, 0.87% K_2_O, 0.44% MgO, 0.56% CaO, and 0.07% TiO_2_, with a loss on ignition 12.31%.

The chosen materials are not commonly used as a backbone material for scaffolds. However, the scope of this work has been precisely to explore the use of porcelains for veneering for ceramic scaffolds. In this respect, it must be highlighted that other ceramic materials have also been tested for manufacturing the samples, for example those that are produced by Ivoclar Vivadent Inc. (Amherst, NY, USA), which are ceramics for dental use as well. Although such a material is in the form of a compact disc (used to produce dental crowns and bridges by milling), it was used as powder to test it for the production of ceramic foams by the template method. However, they did not prove appropriate for this application, as the samples did not have the necessary mechanical resistance for the obtained scaffolds. As a consequence, such samples have been systematically destroyed after burning. Another issue that one might have for other types of commercial ceramics is related to ceramic particles that are too large, therefore, the sponges that are utilized in the method that was applied in this work would not fully absorb the ceramic slurry. *Therefore, one must be careful when considering and processing ceramics for scaffold manufacturing with the method utilized in this work*.

The two commercial ceramics E and D have been already used on the dental market for prosthetic restorations. Thus, they are already approved as biocompatible and bioinert materials, therefore, future in vivo studies to be carried out on animals are going to be much easier to be performed from a legal point of view. This was another criterium for choosing them. These two ceramics exhibit a coefficient of thermal expansion (CTE) that is equal to 12.0 · 10^−6^ K^−1^ (from 20 to 500 °C). For the porcelain-bonding alloy (i.e., material C), the CTE is in the range of 13.8 to 15.1·10^−6^ K^−1^ for a temperature interval from 20 to 500 °C. The firing temperature is 600 °C (1st Dentin), the heating rate is 25 °C/min, the flexural strength equals 90 N/mm^2^, while the leucocyte grain size ranges between 1 and 5 μm.

There were two polyurethane sponges (S.C. Spumotim S.A., Timisoara, Romania) with different densities that were utilized as organic templates for the scaffolds to be manufactured: a green, denser sponge (G), with 300 kg/m**^3^**, which therefore generates a higher density structure, with smaller and fewer pores per volume unit; a blue, less dense sponge (B), with 210 kg/m**^3^**, which therefore generates larger pores and a more porous structure. A characterization of these sponges is made in Section 3.1. *The scaffolds* were made using the foam replication method [27], with a ratio of the ceramic slurry components of 30:70 (% by weight) water: dry material. The manufacturing method (optimized after several trials) involved several steps:

(i) The slurries were prepared by dispersing the ceramic or porcelain powder into distilled water together with a poly-vinylic binder. The porcelain powder morphology is characterized by a certain granulometric distribution, as presented in Section 3.2. The porcelain ceramic slurry was prepared by adding solid porcelain powder 70 wt% to distilled water. The milling process was carried out using a Fritzch Pulverisette planetary mill using porcelain 10 mm balls. As binder, 1.5 wt% Polyvinylalcohol PVA (Sigma Aldrich, Darmstadt, Germany) was added after the milling procedure. This amount is necessary to preserve the slurry stability and to assure the ability of the solid particles to coat the sponge. The ceramic slurries were mixed under magnetic stirring.
(ii) The polymeric sponges were coated with the prepared slurry and then cut to the necessary shape. These sponges were manually immersed in the slurry, and unlike in the traditional replication method, they were not squeezed but kept fully loaded with slurry. The sponges were immediately dried with a multidirectional air flux at 120 °C for 30 min.
(iii) All of the sponges were burned out during a heat treatment which, at the same time, sinters the ceramic powders. Thus, this method has the advantage of obtaining a mass containing an excess of ceramic material; this has a favorable effect on the mechanical resistance of the final product. In consequence, the main aspect which must be discussed—as it may become critical—is related to the porosity of the obtained materials. This process was performed in two steps under atmospheric conditions using an electric furnace (Nabertherm L15/12/P330 Muffle Furnace). The first step which was conducted at 250 °C for 30 min was necessary for burning the sacrificial polyurethane scaffold. In the second step, the ceramic body was sintered at two different temperatures, depending on the precursors that were used: 600 °C for the D and E ceramics, and 900 °C for the novel porcelain C, respectively. The holding time at the sintered temperature was one hour for all of the investigated ceramics, followed by cooling with 100 °C/h until room temperature was reached in order to avoid thermally-induced cracks. Thus, the sponge acted as a template for the porous scaffold.
(iv) Once this polymer sacrificial template was removed, the samples were sintered to the desired structure using an optimized heat-treatment schedule, in two phases: the first one at 250 °C for burning the organic phase; the second one at 600 °C for the D and E ceramics, and at 900 °C for the novel porcelain C, for one hour.

**Figure 1:**
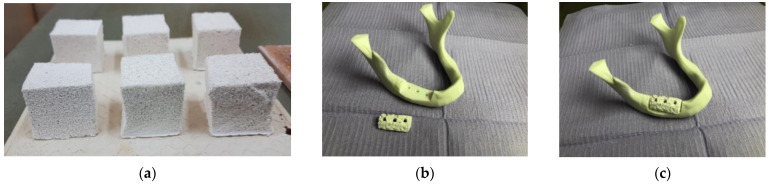
(**a**) Porous ceramic samples that were obtained for the study (cubes with sides of up to 30 mm)—an example from each of the six study groups. An example of the application (with a specific shape of the BC ceramic-based scaffold, manufactured with a different method in [24] (i.e., 3D printing): a mandible (**b**) before and (**c**) after placing the scaffold for bone replacement–as related to our previous study on Finite Element Analysis (FEA) and optimization of the mounting of scaffolds on human mandibles [24]. Further aspects of this application are pointed out in the Section 4.

In all cases, the result was a solid ceramic structure with a precise replica of the geometric characteristics of the polyurethane sponge and with high pores interconnectivity. This polymeric sponge method assured a high thermal and chemical stability, as well as a sufficient hardness of the manufactured samples in comparison to other scaffolding processes [27]. A total of 60 samples divided in six groups were obtained after sintering the three types of ceramic slurry (E, D, and C) on the two types of polyurethane sponge (G and B), as shown in the examples in Figure 1a.

The groups were experimentally named according to the type of ceramic and sponge:

*Group 1 (GE)*: the enamel ceramic (E) is used on the highest density sponge (G).

*Group 2 (BE)*: the enamel ceramic (E) is used on the lowest density sponge (B).

*Group 3 (GD)*: the dental ceramic (D) is used on the highest density sponge (G).

*Group 4 (BD)*: the dental ceramic (D) is used on the lowest density sponge (B).

*Group 5 (GC)*: the new ceramic (C) is used on the highest density sponge (G).

*Group 6 (BC)*: the new ceramic (C) is used on the lowest density sponge (B).

### 2.2. Characterization of the Samples

Several methods were employed to characterize the samples of each group from different points of view, as detailed in the following.

(1) *X-ray diffraction (XRD) analysis.* The phase compositions of the studied ceramic samples were determined with a Rigaku Ultima 4 diffractometer (Rigaku Corp., Tokyo, Japan) using a monochromatic Cu-Kα radiation. The XRD measurements were performed using powdered scaffolds that were obtained after the heat treatment, and dentine and enamel commercial powders for the precursors. All of the samples were scanned over an angular range of 5 to 80° and measurements were recorded using a scanning speed of 20°/min at every 0.05° interval.
(2) *Ascertainment of the apparent porosity*, *p*_ap_, is represented by the ratio between the open pore volume and the apparent volume of the material. It is expressed in % of the latter. In the case of the obtained glass ceramics, the apparent porosity was measured using the liquid saturation method under vacuum, with water as the working liquid. The apparent density [kg/m^3^] is:
  (1) $$\rho_{a}=m/V_{a},$$
  where *m* [kg] represents the mass of the specimen in its dry state; $V_{a}=(m_{1}-m_{2})/\rho_{0}$ [m^3^] is the apparent volume of the specimens, where *m_1_* is the mass of the sample when it is saturated with liquid, *m_2_* is the mass of liquid-weighted specimen, and $\rho_{0}$ [kg/m^3^] is the density of the utilized liquid. Therefore, according to the above definition, the apparent porosity is:
  (2) $$p_{\mathrm{ap}}\;[\%]=\rho_{a}\cdot a/\rho_{0},$$
  where *a* is the absorption capacity of the studied material.
(3) *Scanning electronic microscopy (SEM)* was used to analyze the macro- and micro-porous structure of the obtained ceramics with a Quanta FEG 250 microscope (Thermo Fisher Scientific, Hillsboro, OR, USA) using a low vacuum mode at 3.0 kV.
(4) *Confocal microscopy (CM)* was used to analyze the obtained samples with a LEXT 3D Measuring Laser Microscope OLS4000 (Olympus Europa SE & Co. KG., Hamburg, Germany), which works with a laser centered at a 405 nm wavelength. This scanning microscope is equipped with a confocal system that captures the in-focus image while simultaneously eliminating the flare.
(5) *Mechanical testing* was carried out for each study group using an MTS 643 Compression Plateus (MTS System, Eden Prairie, MN, USA), as shown in the examples that are presented in Section 3. Thus, the compressive strength was determined to assess the capability of the scaffolds to be handled, as well as to perform their functions. Although the standard (i.e., ASTM C1424-15(2019), Standard Test Method for Monotonic Compressive Strength of Advanced Ceramics at Ambient Temperature) recommends cylindrical shape specimens with the length/diameter range from 1.5 to 2.5, we chose cubic specimens for the following technological reasons: (i) the sacrificial polyurethane foam is easier and more precisely cut in this shape compared to the cylindrical shape; (ii) the final ceramic scaffold is easy to be shaped in the desired form starting from cubic form using regular abrasive tools. It should be mentioned that similar specimens were successfully used for compression tests of highly porous alumina-based foam [28].
(6) *Biocompatibility* or cytotoxicity can be verified using two different methods: (i) in vivo tests using human materials, which are too complex and require ethical approvals; (ii) in vitro testing on human or animal cell lines, which is the most common method and it was applied in the present study for the new material C. Biocompatibility was demonstrated in our previous study [25] for the two commercially available materials E and D.

The present research approached the analysis of the biocompatibility/cytotoxicity of Groups GC and BC in the human primary gingival fibroblasts (HGF). The HGF cell line (ATCC^®^ PCS-201-018 ™) was purchased from ATCC (American Type Cell Collection) under the form of frozen vial. The HGF cells are of human origin that were isolated from a Caucasian woman; they are spindle-shaped, adherent, bipolar, and possess refractory properties. The following reagents were used in order to conduct the experiment: specific cell culture environment—Basal fibroblast environment (ATCC PCS-201-030) and Fibroblast growth kit—low serum (ATCC PCS-201-041). The other reagents, i.e., trypsin (EDTA solution), PBS (phosphate saline solution), Trypan blue, and Alamar blue (resazurin sodium salt) were bought from Sigma Aldrich (Darmstadt, Germany) and Thermo Fisher Scientific (USA). The testing compounds GC were dissolved in PBS using ultrasounds. GC—45.55 mg/mL stock solutions were obtained. The HGF cell lines were grown in a specific environment: Medium basal fibroblast (ATCC PCS-201-030) that were supplemented with Fibroblast growth kit—low serum (ATCC PCS-201-041) in cell culture plates T-75. During the experiment, the HGF cells were preserved in a humidified incubator that was supplied with 5% CO_2_ at 37 °C. The cells were counted using Countess^TM^ II automated cell counter (Thermo Fisher Scientific, USA) in the presence of Trypan blue.

In order to evaluate the biocompatibility/cytotoxicity of the compounds that were tested on the HGF cell line, the Alamar blue test was performed. The HGF cells were grown in plates with 96 sample wells (1 × 104 cells/sample well/200 μL) and they were allowed to grow until the corresponding confluence was reached (24 to 48 h). Different concentrations (50, 100, and 250 μg/mL) of test compounds (GC and BC) were added in the fresh cell culture environment and kept for 24 h in contact with the HGF cells. After 24 h, 20 μL of Alamar blue were added, then the cells were incubated for 3 h at 37 °C. The absorption values were measured at 570 and 600 nm with a xMark™ Microplate spectrophotometer (Bio-Rad, Hercules, CA, USA). The cytotoxicity test protocol followed the recommendations that are provided by the ISO-10993-5 standard.

**Figure 2:**
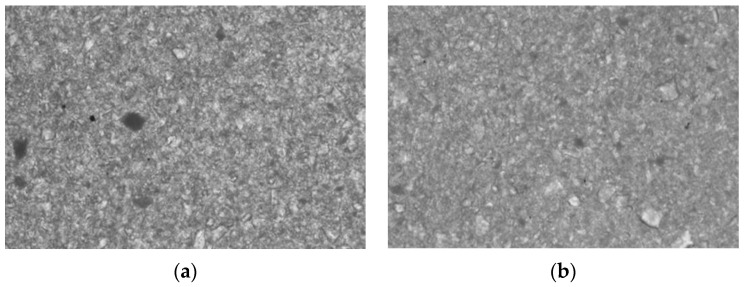
Aspect of ceramic suspensions after solubilization in PBS using ultrasounds: (**a**) Group 5 (GC) and (**b**) Group 6 (BC). The photos were taken using a 20× lens.

To assess the impact of the new ceramic on the HGF viability, the materials were received in the form of solid articles and were solubilized in PBS (i.e., an environment that is biocompatible) by subjecting them to the ultrasonic environment. Figure 2 shows the images of the ceramic suspensions. One can see that they are homogeneous; the particles of distinct colors indicate the different components of the suspensions.

## 3. Results

### 3.1. Sponges Characterization

**Figure 3:**
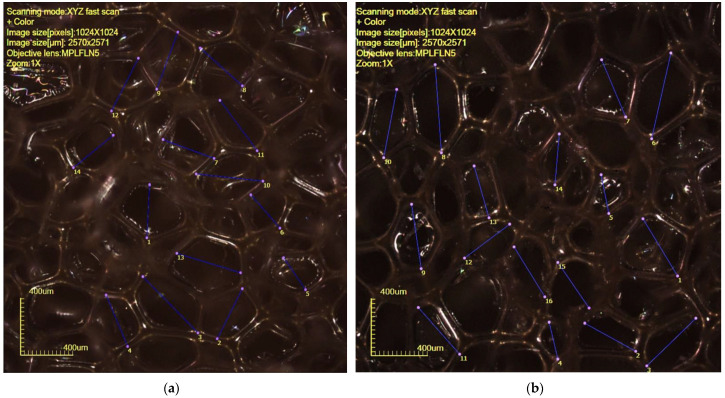
Pore topography for the two polyurethane sponges that were used as sacrificial templates obtained with confocal microscopy: (**a**) green (G) and (**b**) blue (B).

As the two utilized sponges are commercial polyurethane foams, they come without any structural description and no information regarding pore morphology was available for them. The foam density is an informal parameter that could give information regarding the sponges ability to retain the ceramic slurry but not about the pore dimensions and dimensional distribution. Therefore, the sponges surface topography was determined using confocal microscopy, as illustrated in Figure 3.

One can remark on the pore dimensions, with the following parameters that were extracted from these images: a mean of 412 μm and a standard deviation (SD) of 71 μm for the G sponge; a (higher) mean of 446 μm and a SD of 76 μm for the B sponge.

### 3.2. Powder Characterization

**Figure 4:**
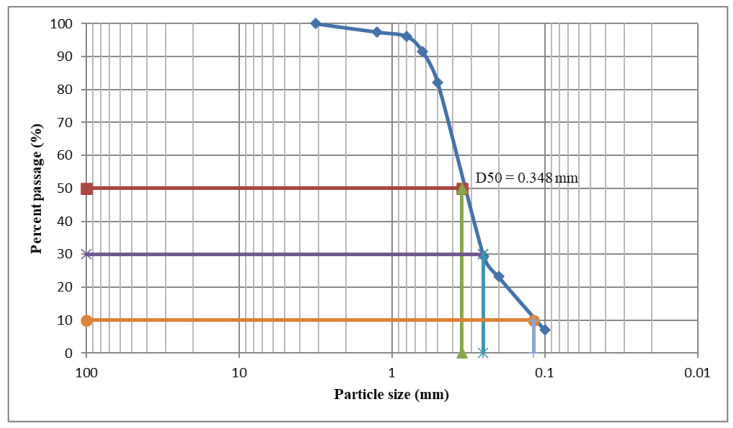
The granulometric distribution that characterizes the porcelain powder morphology.

The porcelain powder morphology that was utilized in the study is characterized by the granulometric distribution presented in Figure 4.

### 3.3. Phase Composition

The obtained samples had an original structure that fits with a (more) resilient outer surface and a porous internal network. This outer surface, which behaves as a ‘shell’ guarantees both high permeability and maneuverability.

**Figure 5:**
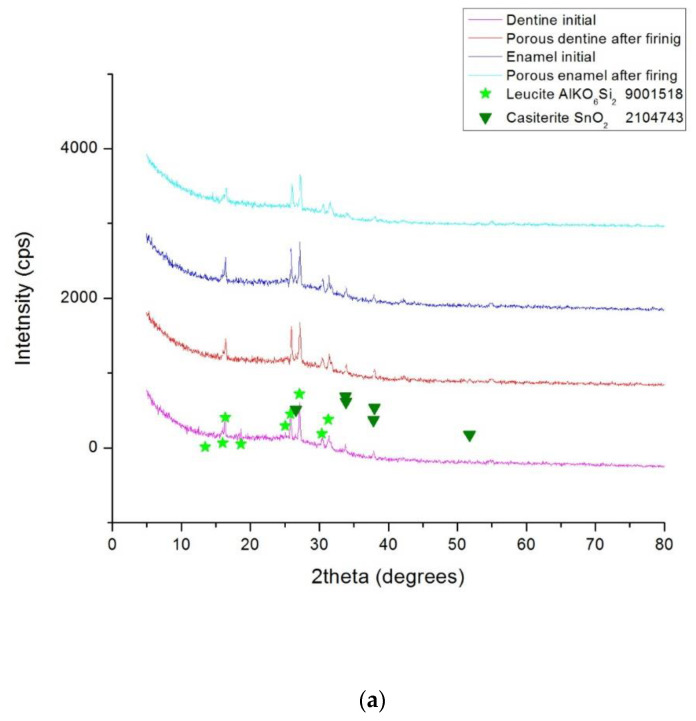

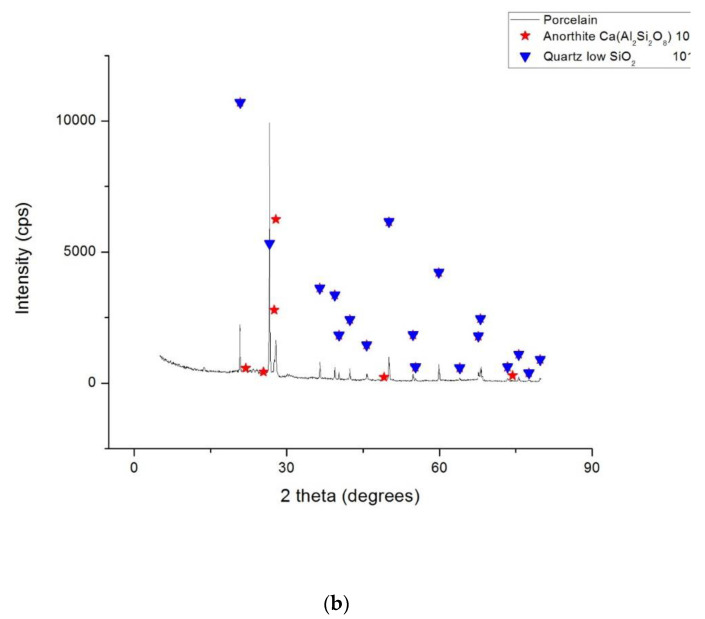
The phase composition of: (**a**) the enamel (E) and the dentin (D) ceramics, corresponding to Groups 1 to 4 (i.e., GE and BE, as well as GD and BD, respectively); (**b**) the new-developed porcelain material—Groups 5 (GC) and 6 (BC).

The chart in Figure 5a shows the phase composition of the dentin (D) and of the email (E) ceramics. The XRD patterns for these two types of samples indicate the presence of the same crystal chemical compounds, both before and after the heat treatment: Leucite (ICDD 15-47) and Cassiterite (ICDD 41-1445). These components correspond to the technical sheet of such materials.

In the case of the novel porcelain (material C), the main phases after firing are Anorthite (ICDD 12-0301) and Quartz (ICDD 33-1161). Both are recognized as biocompatible. The chart in Figure 5b shows the phase composition of this new material.

### 3.4. Apparent Porosities of the Samples

The apparent porosity values that were obtained for the investigated glass ceramic compositions are provided in Table 1. Regarding this parameter, the best results are obtained for ceramic samples falling within the limits that were mentioned in literature for a scaffold to become functional: apparent porosity from 50 to 90%. This was valid especially for the final Group 6 (BC), but also at limit (with almost equal values of the mean) for Groups 4 (BD) and 5 (GC). Significant differences were obtained between all groups (Kruskal–Wallis Test, *p* < 0.001). Also, by comparing the groups in pairs, significant differences were obtained (Mann–Whitney U Test, *p* < 0.001), except for Group 4 (BD) versus Group 5 (GC), which had an insignificant difference (*p* = 0.075).

The obtained samples were similar in terms of morphology to the structure of the natural bone, with a surface (periphery) of the samples (i.e., towards their lower part, where the ceramic material settles more) with a denser structure that is similar to the cortical natural bone, which has a porosity ranging from 3 to 15% [29]. Inside the inner part of the samples their structure is more porous, with both macro- and micro-pores, close to the internal spongy structure of the trabecular bone, which has a porosity of around 80% for the mandibula [30]. The use of dentin D and enamel E from Dentsply Sirona (i.e., the first four study groups in Table 1) led to less porous samples compared to those that were obtained from the new porcelain material C because of the presence of a larger amount of liquid phase that fills the pores at firing temperatures.

### 3.5. Pores Morphology—SEM Investigations

**Figure 6:**
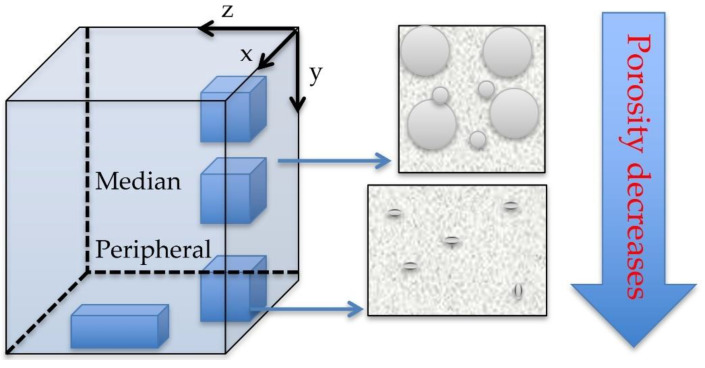
Schematic representation of the porosities in two of the areas to be investigated on the cubic samples in Figure 1a—in relationship with the penetration depth *y* of the ceramic slurry inside the volume of each cube.

Several distinct regions were observed visually on each cubic sample (Figure 1a and Figure 6), but to simplify the discussion, roughly two regions are more clearly distinct:

(m) *The median region* (top and middle part of the cubes) is characterized by a relatively uniform pore distribution with high dimensional scattering;

(p) *The peripheral region* (situated in the lower part of the scaffolds, especially at the bottom, where the ceramic particles have a higher concentration) is characterized by a lower porosity and higher compactness, therefore, it simulates the porosity of the natural bone; this is convenient in the use of the scaffold. The morphology of the porous structure that was obtained is illustrated in the following by using SEM and 3D CM. For both methods, the study was conducted on the two distinct areas above.

**Figure 7:**
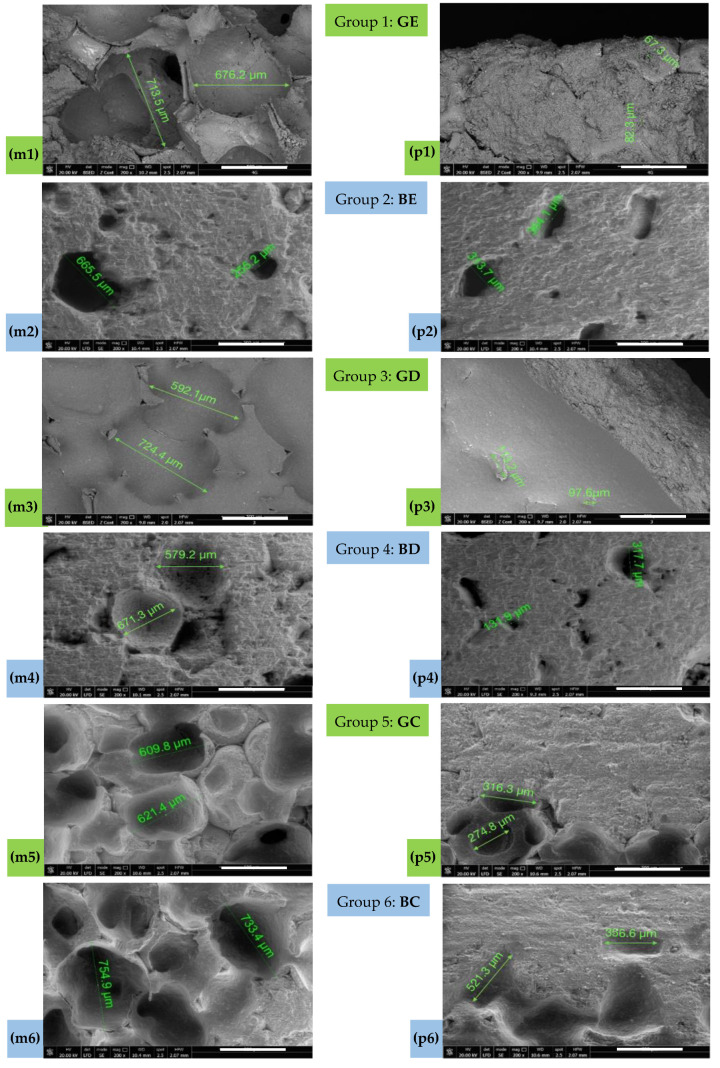
SEM images: (**m1**–**m6**) median/internal and (**p1**–**p6**) peripheral/outer/cortical position for Group **1** (**GE**), Group **2** (**BE**), Group **3** (**GD**), Group **4** (**BD**), Group **5** (**GC**), and Group **6** (**BC**). Scale: 500 μm.

Figure 7 illustrates the macro- and micro-porous structure of the samples, corresponding to the two areas that are mentioned above: an internal (spongy) one and an outer one, cortical, dense. The structure of the internal/median (m) area in all of the samples is composed of macro-pores with dimensions (mainly) between 600 and 750 μm, with the exception of Group 2 (BE), for which there is an even larger distribution. Large cavities are interconnected and there are micro-pores in their inner walls that can be well observed by taking advantage of the high resolution of SEM.

The structure of the cortical/peripheral (p) areas is compact and dense for all of the samples, containing only micro-pores; their purpose is capillary vascularization. However, one can observe the outer surface which has crater-like profiles that resemble macro-pores but are slightly rippled. This is more difficult to observe on the two-dimensional (2D) SEM images, and more convenient to observe on the 3D CM images that are presented in the following subsection.

From the point of view of the spatial and dimensional distribution of the pores, the use of the denser sponge G generated (in contrast to the less dense sponge B) structures with pores of closer size, more evenly distributed in the sample volume (i.e., in the internal areas)—for both enamel E and dentine D.

The situation is different for the material C, for which a more similar internal structure was generated for both G and B sponges, although with a significant increase of the dimensions of the macro-pores for B, as demonstrated by the statistics that are presented in Table 1, as well.

Using the dentin (i.e., Groups 2 (GD) and 3 (BD)), the two studied areas could be distinguished, in contrast to the enamel samples of Group 2 (BE), for which the pores are larger and more evenly distributed in the internal areas. A peculiar case is that of the samples of Group 2 (BE), which apparently are very good and are similar from the SEM images to those of Group 6 (BC). However, this is not true, as demonstrated by the apparent porosity values in Table 1. This shows the limitation (and even the possibility of false results) using SEM, aspect that can be avoided by using CM. However, the clear advantage of SEM is the possibility to observe (and analyze) micro-pores and pore interconnectivity, because of the nanometer resolution of the technique.

From the point of view of the utilized precursor material, there were no clear differences between the samples that were obtained with ceramics E and D. Both materials generated semi-vitrified masses after the heat treatment. The use of the novel porcelain (material C) as a precursor instead of commercially available materials E and D generated a similar structure with regard to the inner and outer areas, but in this case the porous median section consisted of macro-pores of dimensions between 600 and 750 nm for both sponges G and B, with a relatively uniform distribution of these pores in the sample volume.

The results of the measurements that were performed on each sample of the six study groups are presented in Table 2 for the investigations that were performed with SEM. All the samples were prepared in triplicates. The physical measurements for all the investigated material properties were conducted in triplicate. The pore dimensions were measured using the SEM software measurement function. The measured pores diameter is useful to establish a dimensional range for porosity. The dimensional distribution of the pores was not the subject of the investigation.

Significant differences were obtained with the Kruskal–Wallis Test between the results of all the groups, both median (*p* = 0.003) and peripheral (*p* < 0.001). Also, by comparing the groups in pairs, both median and peripheral (Mann–Whitney U Test), the following results were obtained: (i) significantly lower values of Groups 2 (BE), 4 (BD), and 5 (GC) versus Group 1 (GE), (Mann–Whitney U Test, *p* = 0.008); (ii) insignificant difference of Group 3 (GD) versus Group 1 (GE); (iii) significantly higher values of Group 6 (BC) versus Group 1 (GE), (Mann–Whitney U Test, median *p* = 0.016 and peripheral *p* = 0.008), versus Group 2 (BE), (Mann–Whitney U Test, *p* = 0.008), versus Group 3 (GD), (Mann–Whitney U Test, median *p* = 0.032 and peripheral *p* = 0.008), as well as versus Groups 4 (BD) and 5 (GC), (Mann–Whitney U Test, *p* = 0.008).

### 3.6. Confocal Microscopy (CM)

**Figure 8:**
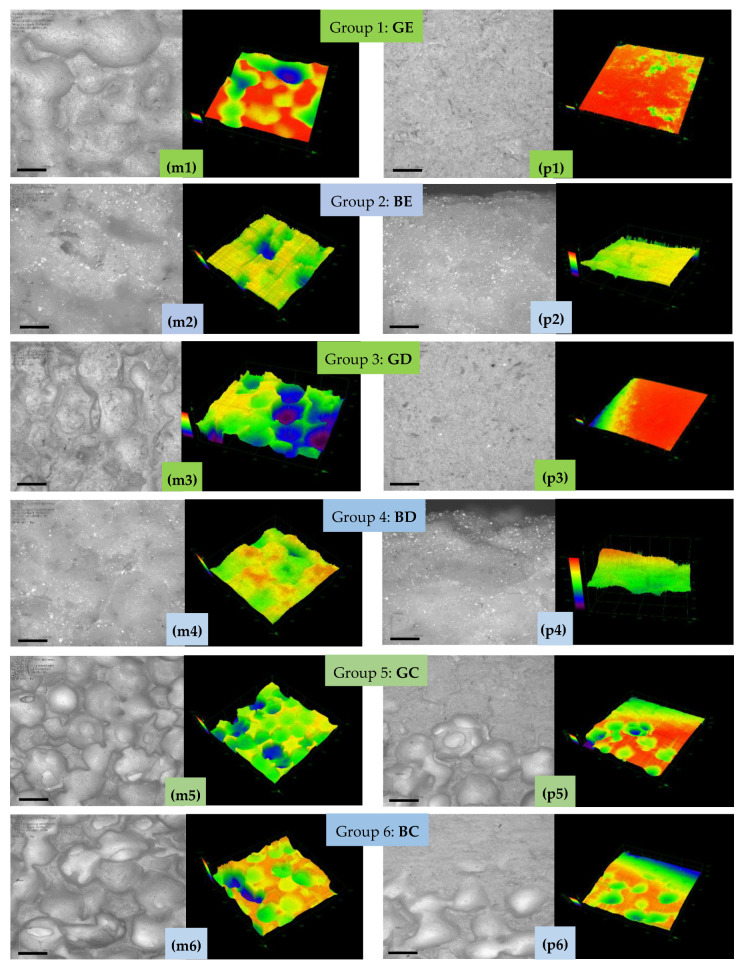
(**m1**–**m6**) Median and (**p1**–**p6**) peripheral/bottom positions for samples from Group **1** (**GE**), Group **2** (**BE**), Group **3** (**GD**), Group **4** (**BD**), Group **5** (**GC**), and Group **6** (**BC**). For each group and considered position, the left image is the microscopic (frontal) one and the right image is the 3D surface reconstruction. Scale: 500 μm.

More intuitive morphological differences between the porous structure of the internal area and the denser structure of the cortical one were obtained using CM/3D laser scanning microscopy (Figure 8).

The lack of pores in the cortical (p) area led to a flatness of this area, while the presence of open pores in the internal (m) area was confirmed by its sponge-like structure in the CM study, presented in Figure 8. In the case of synthesized samples that use the ceramic precursor C (i.e., Groups 5 (GC) and 6 (BC)), a tendency of pore coalescence was observed in the median zone. Such a phenomenon did not appear for the samples of the first four groups that were obtained using enamel E or dentin D. Also, one could observe the porosity difference between the enamel E and the dentin D samples that utilize the more porous polyurethane foam (i.e., the BE and BD samples) and the less porous one (i.e., the GE and GD samples). The former samples presented a better-developed porous structure compared to the latter samples in the median area.

The results of the measurements that were performed on each sample of the six study groups are presented in Table 3 for the investigations performed with CM.

Significant differences were obtained with the Kruskal–Wallis Test between all groups, both median (*p* = 0.012) and peripheral (*p* < 0.001). Also, by comparing the groups in pairs, both median and peripheral (Mann–Whitney U Test), the following results were obtained: (i) for the median zone, insignificant differences of Group 1 (GE) versus Groups 2 (BE), (*p* = 0.690), 3 (GD), (*p* = 0.548), 4 (BD), (*p* = 0.095), 5 (GC), (*p* = 0.151), and 6 (BC), (*p* = 1); (ii) significantly higher values from Group 1 (GE) to Groups 2 (BE), (*p* = 0.008), 3 (GD), (*p* = 0.016), 4 (BD), (*p* = 0.008), 5 (GC), (*p* = 0.008), and 6 (BC), (*p* = 0.008); (iii) in the comparison of Group 6 (BC) versus Group 2 (BE), insignificant differences median (*p* = 1) and significantly higher values peripheral (*p* = 0.008); versus Group 3 (GD), insignificant differences; versus Group 4 (BD), significantly higher values median and peripheral (*p* = 0.016); versus Groups 5 (GC), significantly higher values median (*p* = 0.008) and insignificant differences peripheral (*p* = 0.310).

In all cases (i.e., for all six groups), using both imaging methods, there was a clear distinction between the cortical (p) and the internal (m) structure. The former generated relatively flat surfaces, while the latter, spongy one generated a surface in which open pores could be observed, deep in the bumps.

Using the Mann–Whitney test, all the SEM and CM results (presented in Table 2 and Table 3, respectively) were compared for each of the six study groups. Insignificant differences were obtained, except for the Group 6 (BC), which was characterized by significantly higher mean values in the median zone using SEM compared to CM (Mann–Whitney, *p* = 0.032).

### 3.7. Mechanical Testing Results

**Figure 9:**
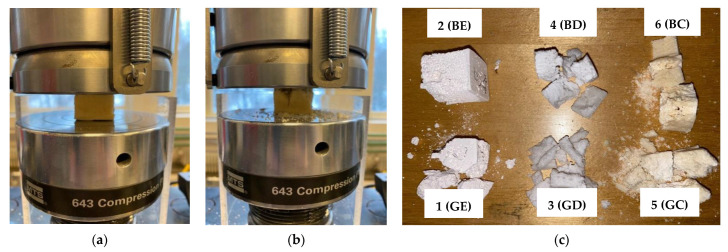
Mechanical testing on the compression force of the developed scaffold samples: (**a**) sample under testing at the (**a**) initial and (**b**) final stage; (**c**) example of a sample from each group that was subjected to compressive testing.

The results of the performed mechanical tests that are shown as examples in Figure 9 are presented in Table 4. The highest values of the compressive strength were obtained for Group 4 (BD), while values that allow the handling of scaffolds were also reached by Groups 6 (BC) and 3 (GD)—to be compared to the porosity results in the following section.

### 3.8. Biocompatibility

**Figure 10:**
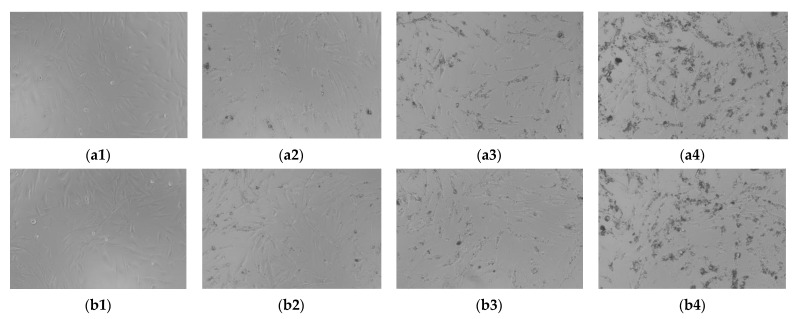
Appearance of primary human gingival fibroblasts (HGF) in culture for 24 h: (**a**) Group 5 (GC) and (**b**) Group 6 (BC), for (**1**) control, (**2**) 50 μg/mL, (**3**) 100 μg/mL, and (**4**) 250 μg/mL. The photos were obtained using the 20× lens.

Changes in cell morphology after contact with a substance are considered specific signs of cytotoxicity. Based on these considerations, the effect that is induced by the types of ceramics that were used in this study for cytotoxicity testing on the morphology of fibroblasts was assessed. As it can be seen in Figure 10, both GC and BC at the lowest tested concentrations (50 and 100 μg/mL) did not change the shape of HGF cells (stimulation for 24 h), compared to the control cells. The cells were adherent to the plate and no reduction in cell growth was reported, which showed a lack of any toxic impact on the cells. For 250 μg/mL, a slight toxicity was concluded for both GC and BC, as there are some cellular residues.

The assessment of the cellular viability was carried out using the Alamar blue test. The difference between the control and the other groups was unidirectionally compared using the ANOVA technique and Dunnett’s post hoc multiple comparative test (GraphPad Prism v. 6.0 Software, San Diego, CA, USA). The difference between the groups was considered statistically significant if *p* < 0.05. The percentage of viable cells compared to the control/unstimulated cells was higher than 95% for the lowest concentrations that were tested (50 and 100 μg/mL) of both GC and BC, showing no cytotoxic effect. It was significantly lower (i.e., 81.94%) only for the BC compound, with a higher value, of 89.12% for the GC compound.

## 4. Discussion

### 4.1. Manufacturing Methods

Ryshkewitch was the first researcher to use a ceramic base on oxide powders to produce portions of alumina and zirconium [31], obtaining porosities of 50 to 60% after a maximum burning temperature of 1850 °C. Similar techniques have used porous hydroxyapatite (HA) bioceramics calcium [32,33] or methyl cellulose as a support for the ceramic that was burned, producing porosities of 60 to 90% [22]. Burning glass ceramic in a stainless-steel mold also produced a high porosity of around 90%. Some methods used custom-made raw materials from calcium phosphate powders, for example with injection molding, but the obtained porosity was low, around 40% [34].

Regarding the inner structure of scaffolds, some methods have been advantageous. Techniques with HA powder samples that were placed in a freezer before sintering produced scaffolds with a relatively uniform structure with unidirectional and parallel pores [35]. Other methods have slight issues, for example using a ceramic bioglass mixture with a polymer binder and foaming agent (subsequently subjected to sintering) produced scaffolds with a low residue content with incomplete crystalline particles [36].

Foam methods are diverse, including materials such as a mixture of ground eggshells and egg white [37], shaken in a controlled way to obtain a foam that was subsequently subjected to heat treatment. In comparison to the above, the method that was developed in this study can have several advantages: (i) it is reproductible, as the properties of the utilized sponges and ceramic materials (or of similar ones) are well-controlled; (ii) some of the materials that were utilized (i.e., ceramics for dental applications) are demonstrated to be biocompatible, therefore, they have the advantage of being already approved for in vivo testing and eventual use; (iii) using sponges can provide scaffolds with a good capability to reproduce the necessary macro-shape that is required by a certain application, as presented in the example in Figure 1b,c; and (iv) the characteristics of the obtained bone scaffolds are appropriate, at least for some of the study groups, as detailed in the following.

### 4.2. Porosity Versus SEM and CM

The optimal characteristics of a functional scaffold according to the literature should include a 50 to 90% apparent porosity, with a minimum pore diameter of 100 μm [38], with the remark that for a successful proliferation of bone tissues, an optimum pore diameter of 200 to 350 μm is necessary [39].

The porosity assessment of the two commercially available sponges that were utilized in this study has been made in Figure 3. However, this is less relevant for the present study compared to the evaluation of the porosity of the obtained scaffolds, which is related to the solid part of the foams. This foam part is destroyed after burning, while the foam pores correspond to the spaces that are filled with slurry during manufacturing.

The apparent porosity analysis of the scaffolds that was designed and manufactured in this work showed that three of the six groups correspond to the above optimal values—slightly higher than 50% for Groups 3 (BD) and 5 (GC) and around 65% for Group 6 (BC)—Table 1. However, this is an average value that characterizes an entire cubic sample, of dimensions of up to 30 × 30 × 30 [mm^3^] considered for each material—Figure 1a. As highlighted by all imaging investigations, (at least) two distinct regions of the samples can be observed. Thus, from Figure 7 and Figure 8, the median regions (m) have an uneven surface with a large scattering of pore depths, while the peripheral regions (p) that are situated at the bottom of the samples (where the ceramic particles sink in higher density) have a more even surface and fewer pores. When implementing the scaffolds—as shown, for example, in the application in Figure 1b,c—this denser, tougher surface is conveniently positioned on top, thus mimicking the outer surface of the bone. One may, therefore, say that the scaffolds that were obtained in Groups 4 (BD), 5 (GC), and especially 6 (BC), (with the higher inner porosity of the latter) are close to natural bone.

A macro-porosity of 150 to 750 μm (with 600 to 750 μm for Group 6 (BC)) was obtained, to allow for waste to dissipate and nutrients to supply the cellular network. In the structure of scaffolds, both macro- and micro-pores must be present, playing a vital role in tissue development. For the developed materials, micro-porosities with a pore size of less than 10 μm, as observed in the high-resolution SEM images in Figure 7, support the development of the smallest capillaries, ensuring vascularization at the lowest cellular level and capillary interactions. Also, the pore distribution should be as uniform as possible across the scaffold structure size to allow for a proper cell attachment and interaction [40], and this is best observed from Figure 7 and Figure 8 for Groups 3 to 6.

An optimal interconnectivity between the pores for the distribution of essential nutrients and oxygen is essential as well. In addition to the pore diameter, other factors such as pore size distribution, pore volume, pore shape, pore neck size, and pore wall roughness should be considered. In particular, data on the interconnected porosity of different macro-porous implants are beneficial to explain the difference in mechanical properties and to differentiate between the advantages and limitations of materials and pore-making methods. Specific surface area (SSA) data can also be evaluated to explain the open or closed macro-pores in the different types of ceramic scaffolds. These aspects are the subject of future work using SEM, micro-CT, and Optical Coherence Tomography (OCT) [41,42,43,44].

### 4.3. Mechanical Properties

Mechanical testing must be performed to make sure that the obtained scaffolds have a sufficient mechanical strength to be handled with ease, a characteristic which is in a trade-off with the as high as possible porosity which should be obtained.

From the point of view of their mechanical properties, biodegradable scaffolds must support the internal architecture of the newly-formed cell matrix. They must assure the mechanical resistance of the tissue that is replaced in the human body, ideally until it is completely resorbed into the body. One must point out that this is different from scaffolds that should not be biodegradable (and thus must target only mechanical performance), in which case materials such as zirconia or alumina are used.

In the literature, the compressive force and traction resistance is about 7 to 10 MPa and 10 to 20 MPa, respectively, for the human trabecular bone, with much higher values of 170 to 193 MPa and 50 to 150 MPa, respectively-for cortical bones [45]. Extending the discussion, all the mechanical properties (i.e., elastic modulus, compressive and tensile strength, maximum strength, and bending modulus) of a scaffold that replaces the bone tissue should have similar properties for a successful therapy. In this respect, the ideal scaffold should have a compression modulus of 10 to 100 MPa [46].

An optimal balance between the porosity of the scaffold that affects the mechanical properties of the material and its mechanical resistance must be obtained, as it was demonstrated that both macro- and micro-pores should be present for an optimal compressive strength of the scaffolds [47]. The SEM investigations in the present study demonstrated that this is the case for all of the developed scaffolds. From Table 4, the compression strength *σ* was higher for Group 4 (BD), of almost 4 MPa, and around 2 MPa for Group 6 (BC). Therefore, in the trade-off between porosity and mechanical characteristics, these results impose BD scaffolds from the point of view of the latter, and BC scaffolds from the point of view of the former.

**Figure 11:**
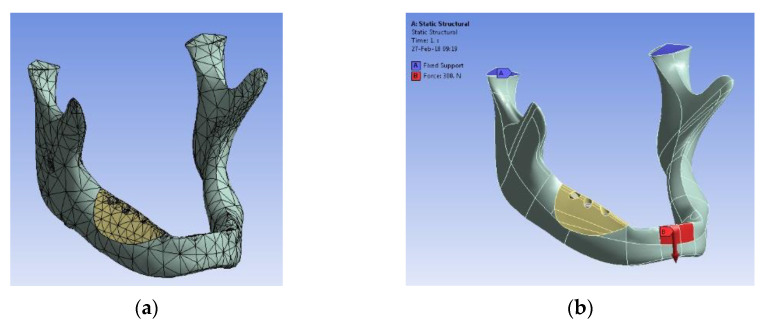

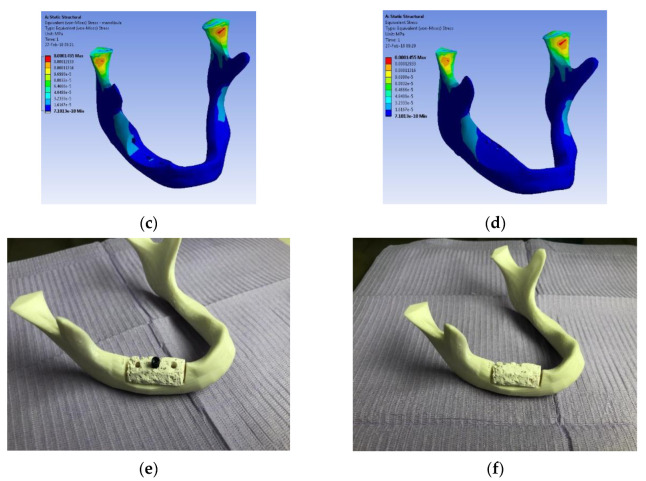
(**a**) Example of an application of ceramic scaffolds such as those that were developed in the present study: FEA performed on a mandible, with (**a**) the design of the mesh, (**b**) application of forces, FEA without (**c**) and with (**d**) the mounted scaffold; physical realization of the assembly, with three (**e**) or with two (**f**) implants—from the detailed study in [24].

While these values of *σ* are not as high as the ideal values above, they prove to be enough for applications with a large biodegradable scaffold such as the one that is presented in Figure 11, because in such a case the masticatory forces do not play a strong role; as the scaffolds are fixed to the (remaining bone of the) mandible using implants and as they have only the role of replacing the missing bone, the stress on their surface is not at the levels that are pointed out for example in [45]. The mechanical properties must also be investigated in wet conditions for applications in the oral cavity, and this is subject of future work.

Such a specific application as the one shown in Figure 1b,c and Figure 11, was extracted from the detailed work in [24], where a FEA was performed for a ceramic scaffold (from the BC material) that was manufactured with a different method (i.e., 3D printing) to be placed on a mandible. The mechanical simulations that were presented with examples in Figure 11a–d and the physical implementations, such as the ones in Figure 11e,f demonstrated the optimal use of such a scaffold, with two (or eventually with three, but with higher costs) implants, as shown in the figure, as well as the capability of such a system to resist to specific forces.

### 4.4. Biocompatibility

*The ability of the material to determine a positive physiological response from the host is another essential requirement of scaffolds, as discussed in the Introduction. The scaffolds materials must be biocompatible* and ideally bioactive, allowing the cells to adhere and proliferate without causing any toxic effects. Also, it should induce the formation of new bone cells [12]. Furthermore, an ideal scaffold should gradually degrade as the new tissue forms in its place [48]. For the E and D materials that were utilized in the present study, biocompatibility was approached in [25], but it is also subject of future work, alongside bioactivity and internal resorption after implementing in the mandible, for example, as shown in Figure 1b,c as well as in Figure 11.

*Biocompatibility* of the new ceramic C was demonstrated for both Groups 5 and 6 (Figure 10), as according to the ISO10993-5 standard, only a compound that induces a viability of less than 70% compared to the control has cytotoxic potential, even if their cytotoxic profile is different. Based on the results that were obtained in Section 3.8, only the BC compound produced a slight toxic reaction at the highest tested concentration (i.e., 250 μg/mL), which was in good agreement with the morphological data in Figure 10. Therefore, we can state that the ceramic product C does not produce any cytotoxic reaction in the human body. Thus, it can easily become a viable solution for the manufacturing of ceramic bone remodeling scaffolds for a prosthetic implant treatment with a high expectation of success.

### 4.5. On the Potential of Other Imaging Methods

Investigations such as the above must be continued ex vivo by taking advantage of the high-resolution capability of micro-CT, which enables the imaging of 3D structures rapidly and non-invasively, giving precise insights herein. In vivo, they could be carried out using OCT, because of its capability to provide most of the information that is offered by methods such as SEM and CM. The limitation that is imposed by OCT resolution (i.e., in the range of micrometers, lower than for SEM, which is in the range of nanometers) allows for observing around 10 μm details of micro-pores, while OCT systems with a 2 μm axial and lateral resolution have been developed as well [49]. In previous studies, we have utilized, for example, 4 to 10 μm axial resolution in-house developed Swept Source (SS) OCT systems to demonstrate that OCT can successfully replace SEM in certain material studies [50,51]. In contrast to other imaging techniques, OCT is non-destructive with regard to materials, and in contrast to SEM and CM it is non-invasive, therefore, it can be applied in vivo, after scaffold implementation. This may leave ex vivo investigations with SEM or CM, for example, but also with the gold standard of histological examination, mainly for validation.

This makes OCT investigation a subject of future work for such applications, especially because of the custom-made 3D shape of scaffolds that must adapt intimately to the defect, to allow for replacing tissue at both microscopic and macroscopic level [52]. This imposes working in situ, with appropriate handheld probes, such as those with 2D galvanometer scanners (GSs) [53], Micro-Electro-Mechanical Systems (MEMS) [49,54], as well as 1D GSs, the latter demonstrated to be capable to satisfy dentistry applications [55,56].

## 5. Conclusions

The study proposed a new ceramic material and developed six types of scaffolds, two of them based on the new material and four of them on commercially available biocompatible materials that have not been utilized so far for such applications. To obtain different porosities, two types of sponges were utilized for each of the three considered materials. The manufacturing process that was based on the foam replication method allowed for obtaining scaffold samples with different porosities. For three out of six study groups, these porosities were higher than the 50% threshold that was pointed out in the literature, while for the final group BC (with the new developed ceramic) the porosity was around 65%. This latter group is characterized by a compressive strength of around 2 MPa, smaller than the ideal values that were pointed in the literature, but which can be included in a scaffold for a mandible, for example, as we explored in a study on mechanical aspects in [24]. Also, one of the groups with a 51% porosity (i.e., BD), was characterized by a compressive strength of around 4 MPa, double than the one above. Therefore, a choice can be made between these two variants of ceramics depending on the most important parameter in this trade-off between porosity and mechanical strength.

From the characterization of the ceramic samples that were obtained in this study with both SEM and CM, the scaffolds that are based on these ceramics are close to the structure of natural bone. SEM demonstrated that the pore morphology is open, and it comprises pores with variable dimensions depending on the area of analysis. Thus, when analyzing the internal surface, the pores are denser on the unit of surface, comprising nano-, micro-, and macro-pores, as it is mentioned in the literature to be optimal in bone augmentation. The external structure of the scaffolds mimics the cortical bone tissue, as it is much denser with a reduced porosity. A strengthening of these features was brought by the 3D CM analysis that presented the same morphological characteristics of the scaffolds and their pores. An important feature that is necessary in bone regeneration through bone augmentation of scaffold type was thus highlighted: open pores exist, and they allow for future blood vessels to develop within the structure. Future work on imaging characterization comprises of high-resolution ex vivo micro-CT investigations, as well as OCT studies, as the latter are non-invasive, therefore, are capable of being utilized in vivo with dedicated handheld probes [53,54,55,56] and systems that are dedicated for dentistry [45].

Following the XRD phase composition analysis, it was shown that the utilized ceramics retain their chemical composition after processing and sintering to obtain scaffolds. Our previous study [25] demonstrated the biocompatibility of scaffolds that were based on the two commercially available ceramics (i.e., E and D) that were included in the present work. The biocompatibility of the new ceramic C was demonstrated in the present study. Future directions of research include comparisons to common materials for biodegradable scaffolds such as calcium phosphates or bioglass.

An exploration of possible manufacturing methods and their optimization is also necessary to further increase the mechanical properties of developed scaffolds in order to bring them closer to the ideal interval of 7 to 10 MPa compressive strength. Also, future work includes applications of such scaffolds for implants in the oral cavity, as approached for the mandible in the preliminary study in [24]. Other issues that must be addressed before clinical use include mechanical reliability, vascular stimulation, and in vivo degradability. The latter allows for avoiding surgical interventions and reduces pain and cost for patients. This can take advantage of the demonstrated properties of bioceramics to form a new bone due to their osteoconductive and sometimes osteoinductive properties.

## Figures and Tables

**Table 1:** The apparent porosity *p*_ap_ [%] for the six study groups.

Group	N	Mean [%]	Standard Deviation [%]	Standard Error	95% Confidence Interval for the Mean		Min.	Max.
					Lower Bound	Upper Bound		
1 **(GE)**	10	29.040	0.391	0.124	28.760	29.319	28.62	29.75
2 **(BE)**	10	45.350	0.573	0.181	44.940	45.760	44.33	45.92
3 **(GD)**	10	32.230	0.589	0.187	31.808	32.652	31.39	32.98
4 **(BD)**	10	**51.260**	0.462	0.146	50.929	51.591	50.53	51.86
5 **(GC)**	10	**51.679**	0.449	0.142	51.358	52.000	50.90	52.30
6 **(BC)**	10	**65.850**	0.621	0.197	65.406	66.295	64.60	66.90

**Table 2:** Ascertainment of the maximum dimension of the pores (μm) on SEM images for the two investigated areas, median (m) and peripheral (p).

Group	Maximum Dimension of the Pores (μm)—SEM													
	Sample 1		Sample 2		Sample 3		Sample 4		Sample 5		Mean		Standard Deviation	
	m	p	m	p	m	p	m	p	m	p	m	p	m	p
1 **(GE)**	713.5	67.3	710	82.3	695.5	77.3	684.9	103.1	709.3	102.7	702.6	86.5	10.79	14.21
2 **(BE)**	665.5	313.7	255.2	364.1	576.2	317.2	679.4	343.1	635.3	307.3	562.3	329.1	157.61	21.33
3 **(GD)**	724.4	97.6	592.1	113.2	671.2	98.3	689.6	107.9	591.6	122.3	653.8	107.9	53.38	9.31
4 **(BD)**	671.3	131.9	673	317.7	602.8	144.6	592.3	272.3	617.7	178.5	631.4	209	34.23	73,27
5 **(GC)**	621.4	316.3	623	274.8	615.7	272.3	624.1	302.9	607.3	283.1	618.3	289.9	6.23	17,03
6 **(BC)**	754.9	521.3	733.4	366.6	711.3	472.2	729.2	476.7	716.2	423.7	729	452.1	15.26	52.75

**Table 3:** Ascertainment of the maximum dimension of the pores (μm) on CM images for the two investigated areas, median (m) and peripheral (p).

Group	Dimension of the Pores (μm)—CM													
	Sample 1		Sample 2		Sample 3		Sample 4		Sample 5		Mean		Standard Deviation	
	m	p	m	p	m	p	m	p	m	p	m	p	m	p
1 **(GE)**	527.4	54.2	773.4	62.4	721.4	60.1	697.4	82.3	659.3	89.8	675.78	69.76	82.9	13.78
2 **(BE)**	634.6	318.2	752.4	275.4	629.6	224.1	702.6	307.2	654.1	296.4	674.66	284.26	46.65	42.57
3 **(GD)**	621.2	83.4	774.5	97.5	754.4	126.4	724.2	92.8	696.4	112.3	714.14	102.48	53.51	15.2
4 **(BD)**	470.4	125.4	642.5	276.5	539.9	208.3	597.3	176.9	608.9	109.5	571.8	179.32	60.54	60.12
5 **(GC)**	603.7	406.8	637.5	213.2	592.3	471.2	616.2	204.7	597.6	262.8	609.46	311.74	15.96	107.73
6 **(BC)**	641.4	341.2	723.1	310.5	670.5	518.4	694.4	273.2	707.3	406.8	687.34	370.02	29.48	86.16

**Table 4:** Results of the mechanical testing that was shown in examples in Figure 9 for the six study groups, with mean values and standard deviation (SD) of the compressive strength.

Group		1 (GE)	2 (BE)	3 (GD)	4 (BD)	5 (GC)	6 (BC)
**Compressive strength *σ* (MPa)**	**Mean**	1.50	0.94	1.57	3.71	1.02	2.09
	**SD**	0.30	0.24	0.16	0.96	0.15	0.36

## References

[1] Langer R., Vacanti J.P. (1993). Tissue Engineering. Science.

[2] Yang S., Leong K.F., Du Z., Chua C.K. (2001). The design of scaffolds for use in tissue engineering. Part I. Traditional factors. Tissue Eng..

[3] Cascalho M., Platt J.L. (2006). The future of organ replacement: Needs, potential applications, and obstacles to application. Transplant. Proc..

[4] Bellucci D., Cannillo V., Sola A. (2010). Shell Scaffolds: A new approach towards high strength bioceramic scaffolds for bone regeneration. Mater. Lett..

[5] Mitragotri S., Lahann J. (2009). Physical approaches to biomaterial design. Nat. Mater..

[6] Matsuno T., Omata K., Hashimoto Y., Tabata Y., Satoh T. (2010). Alveolar bone tissue engineering using composite scaffolds for drug delivery. Jpn. Dent. Sci. Rev..

[7] Francesca G., Alessandro S., Giuseppe M.P. (2013). The biomaterialist’s task: Scaffold Biomaterials and Fabrication Technologies. Joints.

[8] Dhandayuthapani B., Yoshida Y., Maekawa T., Kumar D.S. (2011). Polymeric scaffolds in tissue engineering application: A review. Int. J. Polym. Sci..

[9] Gerecht-Nir S., Radisic M., Park H., Cannizzaro C., Boublik J., Langer R., Vunjak-Novakovic G. (2006). Biophysical regulation during cardiac development and application to tissue engineering. Int. J. Dev. Biol..

[10] Luca R., Todea C.D., Duma V.-F., Bradu A., Podoleanu A. (2019). Quantitative assessment of rat bone regeneration using complex master–slave optical coherence tomography. Quant. Imaging Med. Surg..

[11] Luca R.E., Giuliani A., Mănescu A., Heredea R., Hoinoiu B., Constantin G.D., Duma V.-F., Todea C.D. (2020). Osteogenic Potential of Bovine Bone Graft in Combination with Laser Photobiomodulation: An Ex Vivo Demonstrative Study in Wistar Rats by Cross-Linked Studies Based on Synchrotron Microtomography and Histology. Int. J. Mol. Sci..

[12] Basu B., Nath S., Basu B., Katti D.S., Kumar A. (2009). Fundamentals of Biomaterials and Biocompatibility. Advanced Biomaterials.

[13] Taboas J.M., Maddox R.D., Krebsbach P.H., Hollister S.J. (2003). Indirect solid free form fabrication of local and global porous, biomimetic and composite 3D polymer-ceramic scaffolds. Biomaterials.

[14] Lanza R.P., Langer R., Vacanti J.P. (2000). Principles of Tissue Engineering.

[15] Ma P.X. (2004). Scaffolds for tissue fabrication. Mater. Today.

[16] Griffith L.G., Naughton G. (2002). Tissue Engineering—Current Challenges and Expanding Opportunities. Science.

[17] Deb P., Deoghare A.B., Borah A., Barua E., Das Lala S. (2018). Scaffold Development Using Biomaterials: A Review. Mater. Today Proc..

[18] Bellucci D., Sola A., Cannillo V. (2011). A Revised Replication Method for Bioceramic Scaffolds. Bioceram. Dev. Appl..

[19] Shahgholia M., Olivierod S., Bainob F., Vitale-Brovaroneb C., Gastaldia D., Venaa P. (2016). Mechanical characterization of glass-ceramic scaffolds at multiple characteristic lengths through nanoindentation. J. Eur. Ceram. Soc..

[20] Vargas G.E., Mesones R.V., Bretcanu O., Porto Lopez J.M., Boccaccini A.R., Gorustovich A. (2009). Biocompatibility and bone mineralization potential of 45S5 Bioglass-derived glass–ceramic scaffolds in chick embryos. Acta Biomater..

[21] Ren X., Tuo Q., Tian K., Huang G., Lid J., Xu T., Lv X., Wu J., Chen Z., Weng J. (2018). Enhancement of osteogenesis using a novel porous hydroxyapatite scaffold T in vivo and vitro. Ceram. Int..

[22] Engin N.O., Tas A.C. (1999). Manufacture of Macroporous Calcium Hydroxyapatite Bioceramics. J. Eur. Ceram. Soc..

[23] Appel A.A., Anastasio M.A., Larson J.C., Brey E.M. (2013). Imaging challenges in biomaterials and tissue engineering. Biomaterials.

[24] Gabor A., Zaharia C., Todericiu V., Szuhanek C., Cojocariu A.C., Duma V.-F., Sticlaru C., Negrutiu M.L., Antoniac I.V., Sinescu C. (2018). Adhesion of scaffolds with implants to the mandibular bone with a defect: A Finite Element Analysis. Mater. Plast..

[25] Fabricky M.M.C., Gabor A.-G., Milutinovici R.A., Watz C.G., Avram Ș., Drăghici G., Mihali C.V., Moacă E.-A., Dehelean C.A., Galuscan A. (2021). Scaffold-Type Structure Dental Ceramics with Different Compositions Evaluated through Physicochemical Characteristics and Biosecurity Profiles. Materials.

[26] https://www.dentsplysirona.com/en.

[27] Chen Q.Z., Thompson I.D., Boccaccini A.R. (2006). 45S5 Bioglass^®^-derived glass-ceramic scaffolds for bone tissue engineering. Biomaterials.

[28] Buncianu D., Tessier-Doyen N., Absi J., Negru R., Şerban D.A., Marşavina L. (2020). Multi-Scale Mechanical Behaviour of a Highly Porous Alumina Based Foam. Met. Mater. Int..

[29] Renders G.A., Mulder L., van Ruijven L.J., van Eijden T.M. (2007). Porosity of human mandibular condylar bone. J. Anat..

[30] Cooper D.M.L., Kawalilak C.E., Harrison K., Johnston B.D., Johnston J.D. (2016). Cortical Bone Porosity: What Is It, Why Is It Important, and How Can We Detect It?. Curr. Osteoporos. Rep..

[31] Ryshkewitch E. (1953). Compression strength of porous sintered alumina and zirconia. J. Am. Ceram. Soc..

[32] Klein C.P.A.T., De Groot K., Weiqun C., Yubao L., Xingdong Z. (1994). Osseous substance formation induced in porous calcium phosphate ceramics in soft tissues. Biomaterials.

[33] Yubao L., Klein C.P.A.T., Xingdong Z., De Groot K. (1994). Formation of a bone apatite-like layer on the surface of porous HA ceramics. Biomaterials.

[34] Biggemann J., Pezoldt M., Stumpf M., Greil P., Fey T. (2018). Modular ceramic scaffolds for individual implants. Acta Biomater..

[35] Zhang H., Li X., Wen J., Zhao C. (2017). Preparation and characterisation of HA/TCP biphasic porous ceramic scaffolds with pore-oriented structure. Ceram. Int..

[36] Melli V., Lefebvre L.-P., Lenci M., Mondon M., Sao-Joao S., Cigada A., Delafosse D., De Nardo L. (2017). Resorbability of a Bioglass^®^-based glass-ceramic scaffold produced via a powder metallurgy approach. Ceram. Int..

[37] Sánchez-Salcedo S., Vila M., Diaz A., Acosta C., Barton I., Escobar A., Vallet-Regí M., Synthesis of bioceramic foams from natural products (2017). Mater. Sci..

[38] Rouwkema J., Rivron N.C., van Blitterswijk C.A. (2008). Vascularization in tissue engineering. Trends Biotechnol..

[39] Murphy C., Haugh M., O’Brien F. (2010). The effect of mean pore size on cell attachment, proliferation and migration in collagen-glycosaminoglycan scaffolds for bone tissue engineering. Biomaterials.

[40] Alvarez K., Nakajima H. (2009). Metallic Scaffolds for Bone Regeneration. Materials.

[41] Huang D., Swanson E.A., Lin C.P., Schuman J.S., Stinson W.G., Chang W., Hee M.R., Flotte T., Gregory K., Puliafito C.A. (1991). Optical coherence tomography. Science.

[42] Drexler W., Liu M., Kumar A., Kamali T., Unterhuber A., Leitgeb R.A. (2014). Optical coherence tomography today: Speed, contrast, and multimodality. J. Biomed. Opt..

[43] Hsieh Y.-S., Ho Y.-C., Lee S.-Y., Chuang C.-C., Tsai J.-C., Lin K.-F., Sun C.-W. (2013). Dental Optical Coherence Tomography. Sensors.

[44] Erdelyi R.-A., Duma V.-F., Sinescu C., Dobre G.M., Bradu A., Podoleanu A. (2020). Dental Diagnosis and Treatment Assessments: Between X-rays Radiography and Optical Coherence Tomography. Materials.

[45] Murugan R., Ramakrishna S. (2005). Development of nanocomposites for bone grafting. Compos. Sci. Technol..

[46] Sachlos E., Czernuszka J.T. (2003). Making tissue engineering scaffolds work. Review on the application of solid freeform fabrication technology to the production of tissue engineering scaffolds. Eur. Cells Mater..

[47] Woodard J.R., Hilldore A.J., Lan S.K., Park C.J., Morgan A.W., Eurell J.A.C., Clark S.G., Wheeler M.B., Jamison R.D., Wagoner Johnson A.J. (2007). The mechanical properties and osteoconductivity of hydroxyapatite bone scaffolds with multi-scale porosity. Biomaterials.

[48] Bose S., Roy M., Bandyopadhyay A. (2012). Recent advances in bone tissue engineering scaffolds. Trends Biotechnol.

[49] Cogliati A., Canavesi C., Hayes A., Tankam P., Duma V.-F., Santhanam A., Thompson K.P., Rolland J.P. (2016). MEMS-based handheld scanning probe for distortion-free images in Gabor-Domain Optical Coherence Microscopy. Opt. Express.

[50] Hutiu G., Duma V.-F., Demian D., Bradu A., Podoleanu A.G. (2014). Surface imaging of metallic material fractures using optical coherence tomography. Appl. Opt..

[51] Hutiu G., Duma V.-F., Demian D., Bradu A., Podoleanu A.G. (2018). Assessment of Ductile, Brittle, and Fatigue Fractures of Metals Using Optical Coherence Tomography. Metals.

[52] Sughanthy A.P., Ansari M.N.M. (2015). A Review on Bone Scaffold Fabrication Methods. Int Res. J. Eng. Technol..

[53] Monroy G.L., Won J., Spillman D.R., Dsouza R., Boppart S.A. (2017). Clinical translation of handheld optical coherence tomography: Practical considerations and recent advancements. J. Biomed. Opt..

[54] Lu C.D., Kraus M.F., Potsaid B., Liu J.J., Choi W., Jayaraman V., Cable A.E., Hornegger J., Duke J.S., Fujimoto J.G. (2014). Handheld ultrahigh speed swept source optical coherence tomography instrument using a MEMS scanning mirror. Biomed. Opt. Express.

[55] Demian D., Duma V.-F., Sinescu C., Negrutiu M.L., Cernat R., Topala F.I., Hutiu G., Bradu A., Podoleanu A.G. (2014). Design and testing of prototype handheld scanning probes for optical coherence tomography. J. Eng. Med..

[56] Duma V.-F., Dobre G., Demian D., Cernat R., Sinescu C., Topala F.I., Negrutiu M.L., Hutiu G., Bradu A., Podoleanu A.G. (2015). Handheld scanning probes for optical coherence tomography. Rom. Rep. Phys..

